# Site-Dependent Variation in Phenolics and Antioxidant Capacity of *Berberis vulgaris* Raw Materials: A Multi-Assay Study

**DOI:** 10.3390/antiox15030345

**Published:** 2026-03-09

**Authors:** Asta Mažeikienė, Dovilė Dringelytė, Neringa Burokienė

**Affiliations:** 1 Department of Physiology, Biochemistry, Microbiology and Laboratory Medicine, Institute of Biomedical Sciences, Faculty of Medicine, Vilnius University, M. K. Čiurlionio St. 21, LT-03101 Vilnius, Lithuania; 2 Clinics of Internal Diseases and Family Medicine, Institute of Clinical Medicine, Faculty of Medicine, Vilnius University, M. K. Čiurlionio St. 21, LT-03101 Vilnius, Lithuania

**Keywords:** phenolic compounds, total phenolic content, antioxidant capacity, FRAP, CUPRAC, ABTS, DPPH, geographic variability

## Abstract

Plant phenolics are increasingly explored as natural antioxidants for food systems, yet antioxidant capacity data are difficult to compare because assay chemistry and geographic origin can influence outcomes. Moreover, conventional solvent-based assessments may underestimate the contribution of non-extractable phenolic fractions. Here, *Berberis vulgaris* L. raw materials from six Lithuanian habitats were assessed using hydrolyzed extracts to estimate total releasable phenolics following hydrolytic treatment: total phenolic content (Folin–Ciocalteu) and antioxidant capacity (FRAP, CUPRAC, ABTS, and DPPH) were measured, and fruit extracts were additionally profiled by HPLC–DAD and LC–MS. Across matrices, mean TPC was comparable (108.7 ± 14.1, 111.9 ± 8.4, and 121.9 ± 14.7 mg GAE/g DW for bark, leaves, and fruits, respectively). However, comparable bulk phenolic levels did not translate into uniform antioxidant responses across assays. In contrast, site effects were pronounced, with fruit TPC ranging from 80.0 ± 5.1 to 242.2 ± 61.0 mg GAE/g DW, indicating that geographic origin may outweigh morphological differences when bulk metrics are used. Antioxidant capacity assays further confirmed pronounced site-dependent variability. In particular, leaf extracts exhibited the largest geographic differences, with CUPRAC values ranging from 268.5 ± 32.8 to 586.2 ± 58.6 µmol TE/g DW and ABTS values ranging from 222.0 ± 43.1 to 562.9 ± 26.6 µmol TE/g DW across sampling sites, corresponding to approximately 2.2- and 2.5-fold variation, respectively. Moreover, assay-specific responses led to differences in matrix ranking: bark showed the highest FRAP reducing power (373.2 ± 15.9 µmol TE/g DW), whereas leaves exhibited the highest CUPRAC and ABTS activities (395.7 ± 46.7 and 346.6 ± 48.5 µmol TE/g DW, respectively). Chromatographic profiling of fruits revealed a structurally diverse set of phenolic acids and flavonoids, providing structural support for assay-dependent antioxidant behavior. Overall, integration of multi-assay antioxidant evaluation with hydrolysis-based phenolic assessment and chromatographic profiling provides a broader characterization of *Berberis vulgaris* as a plant matrix of interest for food applications. This integrated approach supports more context-aware interpretation of antioxidant data in applied food research.

## 1. Introduction

Despite the extensive body of literature on plant-derived antioxidants, interpretation of antioxidant capacity data remains challenging due to the intrinsic chemical heterogeneity of phenolic compounds and the strong dependence of analytical outcomes on assay mechanism and sample matrix [1,2,3]. Reported antioxidant activities often vary widely across studies, frequently reflecting methodological choices rather than true differences in bioactive composition [4,5]. This limitation is particularly evident in plants containing multiple redox-active compound classes. In such cases, research emphasis is shaped by dominant constituents rather than by a systematic evaluation of antioxidant-relevant fractions [6].

*Berberis vulgaris* L. exemplifies this issue. The literature on *B. vulgaris* is largely centered on the isoquinoline alkaloid berberine, extensively investigated for pharmacological and clinical relevance, including antimicrobial, antidiabetic, and cardioprotective effects [7,8,9]. While this focus has yielded valuable insights into alkaloid-mediated bioactivities, it has also implicitly framed antioxidant evaluations of *B. vulgaris* through a berberine-centric perspective. Consequently, phenolic compounds—despite their well-established role as major contributors to antioxidant activity in aqueous systems and food matrices—are often treated as secondary constituents rather than as independent determinants of antioxidant behavior [10,11,12]. Moreover, investigations of phenolics in *B. vulgaris* have predominantly focused on solvent-extractable fractions [13,14,15,16,17,18,19,20]. In contrast, total releasable phenolics, including non-extractable or conjugated forms, have received comparatively limited attention in this species. Such fractions have been reported to contribute to total antioxidant capacity in various plant matrices [21].

This imbalance has important methodological and interpretative consequences. Antioxidant capacity attributed to *B. vulgaris* is frequently reported without explicit differentiation between alkaloid- and phenolic-driven effects, even though these compound classes differ substantially in polarity, redox behavior, and analytical response across commonly used antioxidant assays [22,23]. In food science and food processing contexts, where hydrophilic antioxidants are functionally dominant, and matrix effects strongly influence performance, reliance on an alkaloid-oriented framework may obscure the relevance of phenolics and limit the applicability of reported antioxidant data [5,22].

Phenolic compounds are not uniformly distributed within plant tissues but exhibit pronounced plant-part-specific patterns governed by biosynthetic regulation, physiological function, and environmental adaptation [24,25]. Leaves, bark, and fruits thus represent chemically distinct matrices characterized by divergent phenolic profiles and, consequently, distinct antioxidant responses [26,27]. In addition to tissue-specific variability, phenolic biosynthesis is strongly influenced by environmental factors, including light exposure, temperature, soil composition and seasonal variability [27]. Nevertheless, antioxidant studies frequently rely on material collected from a single location, implicitly assuming compositional uniformity and overlooking variability relevant to food quality, standardization, and functional performance [6,23].

The methodological dimension further compounds these challenges. Spectrophotometric antioxidant assays rely on distinct chemical mechanisms, including electron transfer, radical scavenging, and metal ion reduction, each of which responds selectively to specific structural features of phenolic compounds [22,23]. In complex plant matrices, this frequently leads to divergent antioxidant rankings depending on the assay applied, complicating interpretation and cross-study comparison [4,6]. Integrating targeted chromatographic characterization of phenolic compounds with complementary spectrophotometric antioxidant assays provides a mechanistic basis for interpreting antioxidant behavior beyond bulk measurements [28,29]. Although advances in LC–MS-based phenolic profiling have substantially refined structural identification in many plant systems over the past decade, their application to *Berberis vulgaris* phenolics remains comparatively limited and often focused on selected compound groups or single plant organs. A structurally integrated interpretation of antioxidant assay divergence in this species is still insufficiently developed. Taken together, the existing literature on *B. vulgaris* indicates a strong research emphasis on alkaloids, while phenolic investigations have more often focused on solvent-extractable fractions. Consideration of non-extractable phenolic pools remains comparatively limited in this species. In addition, antioxidant assays and chromatographic profiling are frequently reported in parallel, yet less often discussed within an explicitly integrated interpretative framework. Geographically, published phenolic and antioxidant data for *B. vulgaris* are most extensively reported from Türkiye [13,14,15], followed by studies from Central and Southeastern Europe [16,17,18] and Mediterranean regions [19], whereas comparable data from northern Europe (including the Baltic region) are scarce or practically unavailable. This study presents a multi-assay evaluation of antioxidant capacity in *Berberis vulgaris* raw materials collected across multiple natural habitats in a northern European context, with a focus on total releasable phenolics, including non-extractable fractions obtained via sequential hydrolysis. Although the present study did not separately quantify extractable and bound phenolic fractions, the applied sequential hydrolysis protocol enables assessment of the total releasable phenolic pool under standardized analytical conditions, providing a comprehensive estimate of phenolics potentially contributing to antioxidant behavior. Site-dependent variability and tissue-specific phenolic distribution were assessed using complementary spectrophotometric assays and supported by qualitative chromatographic profiling of fruit phenolics by HPLC–DAD and LC–MS. Because antioxidant capacity is not a single physicochemical property, but rather a composite outcome of multiple reaction mechanisms, a multi-assay approach is increasingly recommended to avoid method-dependent bias and to enable matrix-oriented interpretation of results.

## 2. Materials and Methods

### 2.1. Plant Material, Sampling Sites, and Sample Preparation

**Figure 1:**
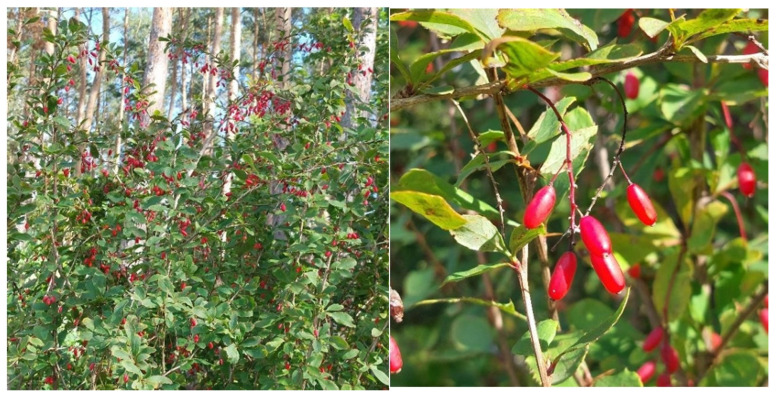
Common barberry (*Berberis vulgaris* L.).

Common barberry (*Berberis vulgaris* L.) (Figure 1) is a deciduous shrub belonging to the barberry family (*Berberidaceae*) and the genus *Berberis*. The family *Berberidaceae* comprises approximately 14–17 genera and about 650–700 species.

Bark, leaves, and fruits of *Berberis vulgaris* L. were collected in Lithuania in 2024 from six distinct locations: (i) Vilnius University Botanical Garden (Kairėnai, Vilnius), and natural habitats including (ii) Trakai district, Totoriškės village; (iii) Kaunas district, Girionys forest; (iv) Jonava district, Guldynai village; (v) Kėdainiai district, Paberžė village; and (vi) Anykščiai district, Pienioniai forest. All sampling sites are located in lowland regions of central and eastern Lithuania and fall within the temperate continental climate zone characteristic of the country. To reduce seasonal variability and ensure comparable physiological stages, bark was collected in early spring (March), leaves after flowering (June), and fully ripe fruits in autumn (October). At each sampling site, plant material was collected to represent the local population and processed as a composite sample. Accordingly, sampling sites were treated as independent biological replicates in the statistical analysis. This approach was adopted to capture site-level variability rather than individual plant-to-plant variation. Collected material was air-dried at room temperature in a well-ventilated area protected from direct sunlight. Dried samples were milled using an analytical mill (A 10 Basic, IKA, Staufen, Germany) and stored in dark glass vials at 4 °C until extraction and analysis.

### 2.2. Chemicals and Reagents

Folin–Ciocalteu reagent (2 M), gallic acid, Trolox, ABTS, DPPH, potassium persulfate, TPTZ, neocuproine, copper(II) chloride dihydrate, iron(III) chloride, sodium carbonate, sodium acetate trihydrate, ammonium acetate, potassium dihydrogen phosphate, disodium hydrogen phosphate, sodium chloride, hydrochloric acid (≥37%), sodium hydroxide, metaphosphoric acid (33.5–36.5%), glacial acetic acid, formic acid, methanol, ethanol, acetonitrile, and deionized water were used. Chromatographic reference standards of phenolic compounds were used for compound identification. Reagents were of analytical grade and purchased from Sigma-Aldrich (St. Louis, MO, USA), Merck (Darmstadt, Germany), Fluka (Buchs, Switzerland), Thermo Scientific (Waltham, MA, USA), and VWR (Radnor, PA, USA).

### 2.3. Instrumentation

Sample preparation and spectrophotometric assays were performed using a UV–Vis microplate reader (Multiskan Sky, Thermo Scientific, Singapore City, Singapore), an analytical balance (KERN PLS 310-3F, Kern&Sohn, Balingen, Germany), vortex mixer (Vortex V-1 plus, BioSan, Riga, Latvia), ultrasonic bath (WUC-A02H, Witeg, Wertheim, Germany), water bath (WB-6, Witeg, Wertheim, Germany), thermostatic incubator (Ecocell 22, MMM Medcenter Einrichtungen GmbH, Munich, Germany), centrifuge (Universal 320R, Hettich, Tuttlingen, Germany), microcentrifuge (Fresco 21, Thermo Scientific, Bremen, Germany), thermostatic shaker (TS-100, BioSan, Riga, Latvia). Chromatographic analyses were performed using an HPLC system equipped with a diode-array detector (L-7450A, Merck Hitachi, Darmstadt, Germany) for UV-based profiling and a liquid chromatography system coupled to a triple quadrupole mass spectrometer (API 2000, AB Sciex, Framingham, Germany) for confirmatory analysis.

### 2.4. Determination of Loss on Drying

Loss on drying (LOD) of dried bark, leaf, and fruit materials was determined according to the European Pharmacopoeia (Ph. Eur. 04/2023:2851) [30]. Briefly, weighing dishes were pre-dried at 105 °C for 30 min, cooled to room temperature, and weighed. Three replicate samples (1.00 g each) were prepared for each plant part. Samples were dried at 105 °C for 2 h, cooled for 30 min, and weighed. Drying/weighing cycles were repeated at 30 min intervals until constant mass was reached (two consecutive weightings differed by ≤0.5 mg).

### 2.5. Preparation of B. vulgaris Extracts (Bark, Leaves, Fruits)

Extracts were prepared based on a published protocol, slightly adapted to the studied matrix [31,32]. To improve recovery of phenolics present as conjugates, samples were subjected to sequential hydrolysis (1.0 M HCl, 2.0 M NaOH, 75% methanol, and 0.75 M metaphosphoric acid; 1 mL each) with intermittent mixing and incubation at 37 °C. This procedure was applied to release phenolic fractions that may not be fully extractable under simple solvent extraction. The aim was to enable comparable assessment of the total releasable phenolic pool across structurally distinct plant matrices (bark, leaves, fruits). After centrifugation, supernatants were collected, and the residue was re-extracted three times with 70% (*v*/*v*) methanol using vortexing and sonication. Combined extracts were adjusted to 25 mL with 70% (*v*/*v*) methanol. Each plant part from each sampling location was extracted in duplicate (*n*_extraction = 2), yielding 36 extracts in total. Prior to assays, extracts were clarified and analyzed at 1:5, 1:10, and 1:20 dilutions (water as diluent) to ensure measurements within each assay’s linear range. Replication scheme: sampling locations were treated as site-level biological replicates (*n*_bio = 6 per plant part). For each extract and dilution, measurements were performed in technical duplicate wells. Although extractions were performed using air-dried plant material, all results were recalculated and expressed per gram of dry weight (DW) based on experimentally determined loss on drying values.

### 2.6. Total Phenolic Content (TPC) by Folin–Ciocalteu Assay

Total phenolic content (TPC) was determined using the Folin–Ciocalteu assay [33] with minor adjustments and in line with standardized reporting practices [1]. The working Folin–Ciocalteu reagent was prepared by diluting the 2.0 M stock 1:10 (*v*/*v*) with deionized water. Sodium carbonate (7.5% *w*/*v*) was prepared by dissolving 3.8 g Na_2_CO_3_ in deionized water and adjusting the volume to 50.0 mL. Gallic acid calibration standards (8.5, 34.0, 85.1, 136.1, and 170.1 mg/L) were prepared in deionized water. In a 96-well plate, 30.0 µL of blank (deionized water), standards, or diluted extracts (1:5, 1:10, 1:20) were combined with 150.0 µL of working Folin–Ciocalteu reagent and 120.0 µL of 7.5% Na_2_CO_3_. Standards were measured in triplicate; samples were measured in duplicate per dilution. Plates were incubated for 2.0 h at room temperature, protected from light, and absorbance was recorded at 740.0 nm after 5.0 min equilibration at 30.0 °C. TPC was calculated from the gallic acid calibration curve and expressed as mg gallic acid equivalents per g dry weight (mg GAE/g DW) using: TPC = (c × V × DF)/m, where c is the gallic acid concentration (mg/L), V is the extract volume (L), DF is the dilution factor, and m is the sample mass (g).

### 2.7. Antioxidant Activity Assays

Antioxidant capacity was evaluated using complementary assays (FRAP, CUPRAC, ABTS, and DPPH), guided by established mechanistic considerations and assay limitations [1,22,23]. Results were expressed as µmol Trolox equivalents per g dry weight (µmol TE/g DW). For each assay run, blanks were used for baseline correction, and standards and samples were analyzed within the linear range.

#### 2.7.1. FRAP Assay

FRAP was performed as described by Benzie and Strain [34]. FRAP reagent consisted of 300 mM acetate buffer (pH 3.6), 10 mM TPTZ in 40 mM HCl, and 20 mM FeCl_3_ mixed at 10:1:1 (*v*/*v*/*v*). In a 96-well plate, 30 µL of blank (deionized water), Trolox standards (12.5–200 µmol/L; triplicates), or diluted extracts (duplicates) were mixed with 200 µL FRAP reagent. Absorbance was recorded at 595 nm after 8 min incubation at 37 °C. Results were expressed as µmol Trolox equivalents per g dry weight (µmol TE/g DW): TE (µmol/g DW) = (c × V × DF)/m.

#### 2.7.2. CUPRAC Assay

CUPRAC was performed as described by Apak et al. [2]. Reagents were 10 mM CuCl_2_·2H_2_O, 1 M ammonium acetate buffer (pH 7), and 7.5 mM neocuproine in ethanol. Each well received 25 µL CuCl_2_, 25 µL neocuproine, and 25 µL buffer. Then 20 µL blank, Trolox standards (6.25–250 µmol/L; duplicates), or extracts (duplicates) were added, followed by 7.5 µL deionized water. After 30 min incubation at room temperature, absorbance was measured at 450 nm. Results were expressed as µmol TE/g DW.

#### 2.7.3. ABTS Assay

ABTS was performed according to Re et al. [35]. ABTS•^+^ radical cation was generated by mixing ABTS solution with potassium persulfate and incubating for 24 h in the dark at room temperature. Prior to analysis, the ABTS working solution was diluted with PBS to obtain an absorbance of 0.70 ± 0.05 at 730 nm. In a 96-well plate, 20 µL of blank, Trolox standards (6.25–250 µmol/L; duplicates), or extracts (duplicates) were mixed with 200 µL ABTS working solution. After 2 min, absorbance was read at 730 nm (30 °C). Results were expressed as µmol TE/g DW.

#### 2.7.4. DPPH Assay

DPPH was performed according to Brand-Williams et al. [36]. A 0.3 mM DPPH solution was prepared in ethanol and stored at 4 °C in the dark. For each sample, 500 µL of diluted extract, blank (70% ethanol), or Trolox standards (12.5–250 µmol/L) was mixed with 500 µL DPPH solution, shaken (1000 rpm, 15 min, 25 °C), and centrifuged (14,000 rpm, 2 min, 19 °C). Then 200 µL was transferred to a 96-well plate, and absorbance was measured at 540 nm. Results were expressed as µmol TE/g DW. The limit of quantification (LOQ) for the DPPH assay was defined as the lowest Trolox-equivalent concentration that could be quantified within the linear calibration range with acceptable precision; operationally, LOQ was calculated as LOQ = 10σ_blank/slope, where σ_blank is the standard deviation of blank measurements, and slope is the slope of the Trolox calibration curve. Extract responses below LOQ were treated as not quantifiable and were excluded from downstream statistical and correlation analyses; accordingly, DPPH responses of bark extracts were below LOQ.

### 2.8. Chromatographic Profiling of Phenolic Compounds

Considering the relevance of *Berberis vulgaris* fruits as a food matrix and the pronounced site-dependent variability observed in antioxidant-related endpoints, qualitative chromatographic profiling was performed on fruit extracts using HPLC–DAD and LC–MS to support interpretation of the spectrophotometric results. Given the chemical complexity of plant-derived matrices, an initial HPLC–DAD screening was employed to comprehensively survey the phenolic profile and to guide the rational selection of candidate compounds for subsequent targeted confirmation by LC–MS. Chromatographic analysis was performed for qualitative characterization of major phenolic compound classes in fruit extracts in order to support spectrophotometric findings, rather than for absolute quantification of individual compounds.

#### 2.8.1. HPLC–DAD Analysis

Chromatographic separation and preliminary identification of phenolic compounds were performed using high-performance liquid chromatography with diode array detection (HPLC–DAD), based on a previously published method [37]. The analysis was applied as a screening step to assess the polyphenolic composition of *Berberis vulgaris* fruit extracts prior to confirmatory LC–MS analysis. Separation was carried out on a reversed-phase Luna C18 column (250 × 4.6 mm, 5 µm; Phenomenex, Aschaffenburg, Germany) maintained at 30 °C, using a diode array detector (L-7450A, Merck Hitachi, Darmstadt, Germany). The injection volume was 50 µL. The binary mobile phase consisted of 0.5% (*v*/*v*) acetic acid in water (solvent A) and methanol (solvent B), delivered at a flow rate of 0.8 mL/min over a total run time of 160 min. Elution was performed using the following gradient program: 0–2 min, 0% B isocratic; 2–6 min, linear gradient from 0% to 15% B; 6–12 min, 15% B isocratic; 12–17 min, linear gradient from 15% to 20% B; 17–35 min, 20% B isocratic; 35–90 min, linear gradient from 20% to 35% B; 90–132 min, 35% B isocratic; 132–150 min, linear gradient from 35% to 80% B; and 150–160 min, linear gradient from 80% to 0% B. The extended gradient program was applied to ensure adequate resolution and structural confirmation of diverse phenolic constituents and was intended for qualitative profiling rather than routine quantitative analysis. DAD detection was performed simultaneously at 254, 280, and 320 nm to enable monitoring of different classes of polyphenolic compounds. Phenolic compounds were tentatively identified based on comparison of retention times and UV–Vis absorbance spectra obtained by diode array detection with those of external reference standards. Compounds detected during this screening step were subsequently subjected to confirmatory identification by LC–MS.

#### 2.8.2. LC–MS (Triple Quadrupole) Analysis

LC–MS analysis was employed for the confirmatory identification of phenolic compounds previously detected during HPLC–DAD screening of *Berberis vulgaris* fruit extracts. Analyses were performed using a triple quadrupole mass spectrometer (API 2000, AB Sciex, Darmstadt, Germany). The analytical conditions were adapted from a previously published method [38] with minor modifications to account for the investigated plant matrix. Chromatographic separation was achieved on a reversed-phase Kinetex C18 column (150 × 2.1 mm, 5 µm; Phenomenex, Aschaffenburg, Germany) fitted with a C18 guard column (30 × 4.0 mm) and maintained at 30 °C. The mobile phase consisted of 0.3% (*v*/*v*) formic acid in water (solvent A) and acetonitrile (solvent B), delivered at a gradient elution program as follows: 0–2 min, 90% A/10% B; 12 min, 80% A/20% B; 22–25 min, 78% A/22% B; 27–38 min, 90% A/10% B, resulting in a total run time of 38 min. UV detection was carried out at 254 nm. Mass spectrometric detection was performed in negative ion mode using atmospheric pressure chemical ionization (APCI). Nitrogen was used as the curtain and nebulizer gas. The source temperature was set to 340 °C. Instrumental parameters were as follows: curtain gas 50 psi, nebulizer gas (Gas 1) 80 psi, auxiliary gas (Gas 2) 10 psi, needle current −1 µA, declustering potential −20 V, focusing potential −350 V, and entrance potential −7 V. Compound identification was based on the comparison of retention times and mass-to-charge (*m*/*z*) ratios with those of authentic reference standards analyzed under identical LC–MS conditions.

### 2.9. Statistical Analysis

Data were processed using Microsoft Excel 2021 and IBM SPSS Statistics 30. Data visualization and figure preparation were performed using Python 3.11 with the matplotlib library (version 3.8.2). Variability within datasets was evaluated using coefficients of variation and graphical inspection.

Normality of data distribution was assessed prior to statistical testing using graphical inspection and the Shapiro–Wilk test. No data transformations were applied, as variables meeting the assumptions for parametric testing were analyzed directly. Group comparisons were conducted using one-way analysis of variance (ANOVA) for normally distributed data or the Kruskal–Wallis and Mann–Whitney U tests for non-normally distributed data, as appropriate. When applicable, post hoc pairwise comparisons were performed. Results for normally distributed variables are presented as mean ± standard deviation (SD), whereas non-normally distributed variables are reported as median and interquartile range (IQR). Differences were considered statistically significant at *p* < 0.05.

Associations between total phenolic content and antioxidant activity were evaluated using Spearman correlation coefficients. All analytical results were normalized to dry weight (DW) using experimentally determined loss on drying values to ensure accurate comparison among plant parts and sampling sites.

For each assay, technical replicate wells were averaged for each extract dilution prior to calculation of concentration equivalents. Two independent extraction replicates were prepared for each plant part at each sampling site (*n*_extraction = 2) and averaged to obtain a single value per site, which was treated as a biological replicate. Accordingly, statistical analyses across plant parts and sampling sites were performed using *n* = 6 biological replicates per plant part (sampling sites), unless otherwise stated (e.g., DPPH values for bark extracts were below the limit of quantification and excluded from statistical analyses).

## 3. Results

### 3.1. Loss on Drying of Berberis vulgaris Raw Materials

Loss on drying differed among the plant parts investigated (Table 1). Fruits exhibited the highest moisture content (9.8 ± 0.2%), followed by leaves (7.6 ± 0.8%), whereas bark showed the lowest value (5.5 ± 0.9%). The moisture content of bark was approximately 1.7-fold lower than that of fruits. All samples complied with the European Pharmacopoeia requirement for herbal drugs and did not exceed 10% (*w*/*w*) [30].

### 3.2. Total Phenolic Content in Bark, Leaf, and Fruit Extracts

Total phenolic content (TPC), determined by the Folin–Ciocalteu method in hydrolyzed methanolic extracts and expressed as milligrams of gallic acid equivalents per gram of dry weight (mg GAE/g DW), did not differ significantly among plant parts (*p* > 0.05). Mean TPC values tended highest in fruit extracts (121.9 ± 14.7 mg GAE/g DW), followed by leaf extracts (111.9 ± 8.4 mg GAE/g DW) and bark extracts (108.7 ± 14.1 mg GAE/g DW). The lowest variability was observed in leaf extracts (CV = 7.5%), whereas fruit and bark extracts exhibited comparable variability (12% and 13%, respectively) (Table 2).

It should be noted that the Folin–Ciocalteu assay provides an estimate of the overall reducing capacity of the extracts and is not fully specific to phenolic compounds; therefore, the term “total phenolic content” is used operationally throughout this study and interpreted in conjunction with complementary antioxidant assays and chromatographic data.

### 3.3. Geographic Variation in Total Phenolic Content

TPC varied significantly depending on the geographic origin of the plant material (Table 3). In bark extracts, TPC ranged from 88.3 ± 7.9 to 124.0 ± 9.7 mg GAE/g DW. The highest values were observed in samples collected in Girionys forest (Kaunas district) and Paberžė village (Kėdainiai district), whereas the lowest TPC was detected in Pienionys forest (Anykščiai district) (*p* < 0.05).

Leaf extract TPC ranged from 81.1 ± 8.1 to 166.9 ± 7.9 mg GAE/g DW. Samples from Girionys forest (Kaunas district) exhibited significantly higher TPC compared to other sampling locations (*p* < 0.05). Fruit extracts showed the widest variation in TPC, ranging from 80.0 ± 5.1 to 242.2 ± 61.0 mg GAE/g DW.

The highest TPC was detected in fruits collected in Guldynai village (Jonava district), whereas the lowest was observed in Pienionys forest (Anykščiai district), corresponding to an approximately three-fold difference (*p* < 0.05).

### 3.4. Antioxidant Activity of Extracts

Antioxidant activity of hydrolyzed methanolic extracts was evaluated using the FRAP, CUPRAC, and ABTS assays (electron-transfer-based methods), while radical scavenging activity was determined using the DPPH assay. All results are expressed as micromoles of Trolox equivalents per gram of dry weight (µmol TE/g DW) (Table 4).

**Figure 2:**
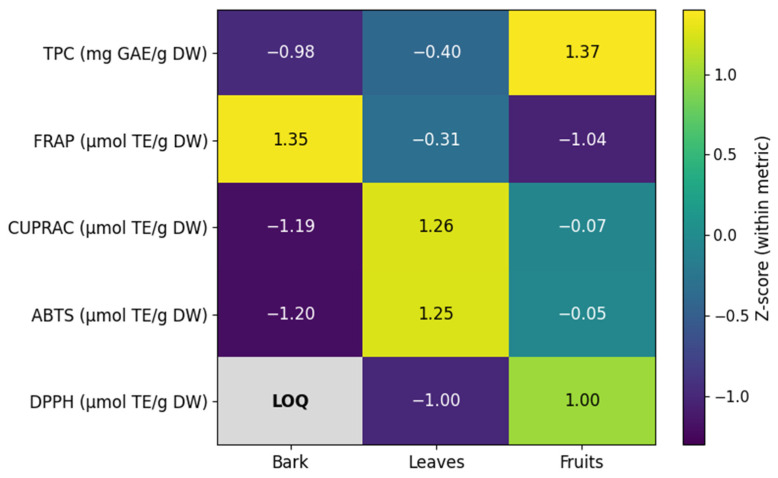
Z-score–normalized heatmap summarizing the mean total phenolic content (TPC) and antioxidant assay responses across sampling sites and *Berberis vulgaris* raw materials. Values were standardized within each variable (row) to enable comparison of relative patterns across assays and plant parts; colors indicate deviation from the variable mean (Z-score). DPPH values for bark extracts were below the limit of quantification (LOQ) and are shown as not quantifiable.

Differences in antioxidant capacity among plant parts are further illustrated in a normalized heatmap summarizing mean TPC and antioxidant assay responses across plant parts (Figure 2).

#### 3.4.1. Ferric Reducing Antioxidant Power (FRAP)

FRAP values differed significantly among plant parts (*p* < 0.05). Bark extracts exhibited the highest mean reducing capacity (373.2 ± 15.9 µmol TE/g DW), followed by leaf extracts (305.7 ± 32.3 µmol TE/g DW) and fruit extracts (275.9 ± 26.7 µmol TE/g DW). Across geographic locations, FRAP values ranged from 276.7 ± 18.2 to 463.1 ± 20.6 µmol TE/g DW in bark extracts, from 206.9 ± 15.7 to 487.7 ± 27.7 µmol TE/g DW in leaf extracts, and from 208.4 ± 19.3 to 340.8 ± 34.0 µmol TE/g DW in fruit extracts. Overall, higher FRAP values tended to be observed in samples collected in Girionys forest (Kaunas district).

#### 3.4.2. Cupric Reducing Antioxidant Capacity (CUPRAC)

CUPRAC activity also differed significantly among plant parts (*p* < 0.05), following a different trend compared with FRAP. Leaf extracts exhibited the highest mean CUPRAC activity (395.7 ± 46.7 µmol TE/g DW), followed by fruit extracts (316.2 ± 32.5 µmol TE/g DW), whereas bark extracts showed the lowest values (248.9 ± 24.5 µmol TE/g DW). CUPRAC values ranged from 199.3 ± 22.1 to 307.3 ± 51.2 µmol TE/g DW in bark extracts, from 268.5 ± 32.8 to 586.2 ± 58.6 µmol TE/g DW in leaf extracts, and from 232.0 ± 25.0 to 403.5 ± 27.4 µmol TE/g DW in fruit extracts. The highest CUPRAC activity was consistently detected in samples from Girionys forest (Kaunas district).

#### 3.4.3. ABTS Radical Cation Scavenging Activity

ABTS radical scavenging activity differed significantly among plant parts (*p* < 0.05). Leaf extracts demonstrated the highest mean activity (346.6 ± 48.5 µmol TE/g DW), followed by fruit extracts (292.4 ± 28.0 µmol TE/g DW) and bark extracts (244.6 ± 27.9 µmol TE/g DW). Across sampling sites, ABTS activity ranged from 186.6 ± 21.2 to 299.2 ± 33.8 µmol TE/g DW in bark extracts, from 222.0 ± 43.1 to 562.9 ± 26.6 µmol TE/g DW in leaf extracts, and from 212.4 ± 23.8 to 378.7 ± 42.3 µmol TE/g DW in fruit extracts. The highest ABTS activity was observed in leaf extracts collected in Girionys forest (Kaunas district).

#### 3.4.4. DPPH Radical Scavenging Activity

DPPH radical scavenging activity was determined in methanolic extracts of leaves and fruits. The operational limit of quantification (LOQ) was defined as the lower limit of the validated Trolox calibration range (12.5 µmol/L), corresponding to approximately 10% inhibition under the applied assay conditions. Responses below this calibration threshold were reported as below LOQ. Fruit extracts exhibited a higher mean activity (243.4 ± 56.6 µmol TE/g DW) compared with leaf extracts (205.9 ± 84.9 µmol TE/g DW), and the difference between plant parts was statistically significant (*p* < 0.05). DPPH absorbance values obtained for bark extracts were below the LOQ under the applied analytical conditions and therefore were excluded from further statistical and correlation analyses. Leaf extract DPPH activity ranged from 137.5 ± 27.9 to 269.5 ± 153.4 µmol TE/g DW and did not differ significantly among sampling locations (*p* > 0.05). In contrast, fruit extract DPPH activity ranged from 156.8 ± 33.6 to 308.7 ± 55.2 µmol TE/g DW and showed significant site-dependent differences (*p* < 0.05).

### 3.5. Qualitative Profiling of Phenolic Compounds in Fruit Extracts

Qualitative chromatographic profiling of *Berberis vulgaris* fruit extracts was carried out using HPLC–DAD followed by LC–MS. HPLC–DAD served as an initial screening step, allowing tentative assignment of several phenolic compounds based on retention times and UV–Vis spectra in comparison with reference standards. Subsequent LC–MS analysis confirmed selected phenolic acids and flavonoids by matching retention times and mass-to-charge (*m*/*z*) values to authentic standards, while additional constituents were retained as tentative assignments supported by the HPLC–DAD data. Overall, the fruit extracts exhibited a structurally diverse phenolic composition. Identified phenolic acids included gallic, chlorogenic, caffeic, and syringic acids, representing both hydroxybenzoic and hydroxycinnamic subclasses. The flavonoid fraction comprised flavan-3-ols (catechin, proanthocyanidin B2), flavonoid glycosides (quercitrin), flavonol glycosides (rutin), and the flavonoid aglycone quercetin. Selected compounds (e.g., chlorogenic acid, caffeic acid, catechin, quercitrin, quercetin) were confirmed by LC–MS, whereas others were tentatively assigned based on HPLC–DAD retention behavior and UV–Vis spectral characteristics. The complete summary of detected compounds and identification approaches is presented in Table 5. Representative HPLC–DAD chromatograms acquired at different detection wavelengths, together with LC–MS extracted-ion chromatograms (XICs), are provided in the Supplementary Materials to illustrate compound identification across different phenolic classes.

### 3.6. Correlation Between Total Phenolic Content and Antioxidant Activity

Given the compositional complexity of plant matrices, total phenolic content was evaluated as a bulk indicator, while antioxidant capacity was interpreted using multiple complementary assays reflecting different reaction mechanisms. Spearman correlation analysis revealed statistically significant positive associations between TPC (mg GAE/g DW) and antioxidant activity (µmol TE/g DW), with correlation strength depending on plant part and assay (Table 6).

In bark extracts, strong correlations were observed between TPC and FRAP (rs = 0.72), CUPRAC (rs = 0.76), and ABTS (rs = 0.82) (*p* < 0.05). In leaf extracts, very strong correlations were found for FRAP (rs = 0.90), CUPRAC (rs = 0.93), and ABTS (rs = 0.95), whereas the correlation with DPPH was weak (rs = 0.33). In fruit extracts, moderate correlations were observed for FRAP (rs = 0.51), CUPRAC (rs = 0.63), and ABTS (rs = 0.54), and a weak correlation for DPPH (rs = 0.35) (*p* < 0.05).

Overall, these results indicate that higher total phenolic content is associated with higher antioxidant activity, particularly in electron-transfer-based assays, while DPPH responses show weaker coupling with TPC.

## 4. Discussion

### 4.1. Loss on Drying and Sample Quality

Loss on drying (LOD) values obtained for *Berberis vulgaris* bark, leaves, and fruits were below the maximum limit of 10% established by the European Pharmacopoeia, confirming adequate drying conditions and suitability of the plant material for quantitative phytochemical analysis [30]. Comparable LOD ranges for *B. vulgaris* raw materials have been reported previously, indicating that moisture content is not a limiting factor in phenolic extraction when standardized drying protocols are applied. Proper control of residual moisture is particularly important for phenolic compounds, as excessive water content may promote enzymatic degradation and reduce extraction efficiency.

### 4.2. Total Phenolic Content in Different Plant Parts

The present study demonstrated that total phenolic content (TPC) varied among *B. vulgaris* plant parts, with fruits tending to exhibit higher mean TPC, followed by leaves and bark. Although differences between plant parts were not statistically significant when averaged across sites, substantial variability was observed depending on geographical origin. The tendency toward higher TPC in fruits is consistent with their biological role as protective tissues enriched in phenolic acids, flavonoids, and proanthocyanidins, which contribute to defense against oxidative stress and herbivory [39]. However, organ-level trends reported for *B. vulgaris* differ across studies, and absolute TPC values vary considerably depending on extraction strategy and expression basis, which complicates direct cross-study comparison.

Recent literature further highlights this methodological heterogeneity in reported TPC values for *B. vulgaris*. Most studies employing conventional solvent-based extraction without hydrolysis report markedly lower bulk TPC values than those observed in the present work. For instance, Iskender et al. [14] reported 27.65 ± 1.12 mg GAE/g in fruits and 20.17 ± 1.24 mg GAE/g in leaves, while Gidic et al. [15] reported 15.37–28.92 mg GAE/g in cultivated and wild fruits. Similarly, Nova-Baza et al. [40], in a multi-species comparison including *B. vulgaris*, reported 21 ± 2 mg GAE/g DW in leaves. However, not all recent solvent-based investigations yield lower values. Dimitrijević et al. [17] reported comparatively higher TPC values despite applying conventional extraction, further underscoring the pronounced methodological and matrix-dependent variability in bulk TPC estimates across studies.

The sequential acid–alkaline hydrolysis applied in the present study was designed to assess total releasable phenolics under standardized analytical conditions. In many plant matrices, a substantial proportion of phenolic compounds occurs in conjugated or insoluble-bound forms associated with structural components of the cell wall [41]. Hydrolytic treatment enables release of these non-extractable fractions and may influence phenolic integrity [42], thereby in many cases, contributing to higher bulk TPC values compared with solvent extraction alone; however, reported TPC values remain strongly influenced by plant matrix, extraction conditions, and expression basis. Accordingly, the values reported here reflect total releasable phenolics rather than intact native phenolic profiles, and cross-study comparisons should be interpreted with methodological caution.

### 4.3. Influence of Geographical Origin on Phenolic Content

Marked differences in TPC among sampling sites were observed across all plant parts, with the most significant variability observed in fruits. In particular, fruits collected from the Kaunas and Jonava districts exhibited substantially higher TPC values than those from other Lithuanian locations. Notably, these two districts are geographically close, suggesting that even relatively small-scale environmental heterogeneity may substantially influence phenolic accumulation. Environmental factors such as temperature, solar radiation, soil composition, and water availability are known to modulate phenolic biosynthesis by regulating the phenylpropanoid pathway [43]. Consequently, cumulative local environmental influences may alter the synthesis and relative abundance of phenolic acids, flavonoids, and related compounds, leading to measurable shifts in bulk TPC values.

The magnitude of site-dependent variability observed in the present work is consistent with differences reported across origins in recent studies. For example, Eroğlu et al. [13] reported wide variation in total phenolic content among wild *B. vulgaris* fruit samples collected within a single Turkish province, with values spanning approximately 148.0 ± 30.3 to 448.3 ± 81.2 µg GAE/mg DM depending on sampling site and extraction solvent. Such findings indicate that pronounced geographic variability may occur even within relatively confined regions.

At the same time, cross-study comparison of geographic effects in *B. vulgaris* remains challenging, as reported absolute values are strongly influenced by extraction strategy, hydrolytic treatment, and reporting basis. Differences between studies may therefore reflect both biological variability and methodological diversity. In contrast, the present study applied a standardized hydrolysis-based protocol and unified analytical framework across all sampling sites, allowing site-dependent differences to be interpreted within a controlled methodological context. The present data extend the available geographic range of phenolic characterization to northern European populations, which remain comparatively underrepresented in the current literature.

Together with the present findings, these data support the notion that geographic origin can drive substantial shifts in bulk phenolic estimates, and that such shifts may be comparable in magnitude to, or exceed, differences attributable solely to plant organ when bulk metrics are used.

### 4.4. Antioxidant Activity Assessed by Electron Transfer-Based Assays

Antioxidant activity determined by FRAP, CUPRAC, and ABTS assays revealed clear differences among plant parts and sampling sites. Bark extracts exhibited the highest reducing power in the FRAP assay, whereas leaves showed the highest activity in CUPRAC and ABTS assays. This discrepancy reflects differences in the underlying reaction mechanisms of these assays and the chemical nature of the dominant antioxidant compounds [1].

FRAP is particularly sensitive to compounds capable of reducing ferric ions under acidic conditions [34], such as phenolic acids and condensed tannins, which are commonly associated with bark tissues. In contrast, CUPRAC and ABTS assays operate at near-neutral pH [2,35] and are more responsive to a broader range of flavonoids and phenolic glycosides, which are prevalent in leaves. Recent literature further illustrates the pronounced variability of electron transfer-based antioxidant values in *B. vulgaris*, depending on extraction strategy and reporting format. For example, Moldovan et al. [18] reported FRAP values of 302.46 ± 15.26 mg TE/g DW and ABTS values of 30.98 ± 0.65 mg TE/g DW in solvent-extracted material. Gidic et al. [15] observed FRAP values ranging from 349.52 ± 1.49 to 621.02 ± 25.03 µmol FeSO_4_/g in fruit extracts, highlighting variability associated with plant origin and cultivation status. Nova-Baza et al. [40] reported ABTS and CUPRAC values of 118 ± 18 µmol TE/g DW and 284 ± 50 µmol TE/g DW, respectively, in *B. vulgaris* leaves, further demonstrating assay-dependent divergence within the same plant matrix.

In addition, Iskender et al. [14] expressed antioxidant capacity using assay-specific units (CUPRAC: 0.203 mmol TEAC/g; FRAP: 0.15 mmol Fe/g in fruit extracts), underscoring how differences in analytical units and extraction conditions complicate direct numerical comparison across studies. Taken together, these data confirm that electron transfer-based antioxidant capacity in *B. vulgaris* is strongly method-dependent and highly sensitive to both extraction protocol and reporting basis. Importantly, although hydrolysis-based protocols are sometimes assumed to substantially elevate antioxidant estimates, evaluation of the present results in the context of recent solvent-extraction studies indicates that antioxidant values in *B. vulgaris* remain highly scattered across the literature regardless of extraction strategy. In several reports, solvent-extracted samples exhibited comparable or even higher FRAP or DPPH values than those observed under the present hydrolysis-based conditions. However, direct numerical comparison across studies performed under different extraction protocols, assay conditions, and reporting formats is inherently limited. These observations suggest that inter-study variability cannot be attributed solely to hydrolytic release of bound phenolics, but rather reflects the combined influence of plant origin, matrix composition, analytical conditions, and expression basis.

### 4.5. Radical Scavenging Activity and Method-Dependent Differences

DPPH radical scavenging activity was detected only in leaf and fruit extracts, while bark extracts exhibited values below the limit of quantification under the applied analytical conditions. This observation reflects assay sensitivity and matrix-dependent response characteristics rather than the absence of radical-scavenging compounds. Compared with leaves and fruits, bark matrices may contain a higher proportion of structurally complex or polymerized phenolic constituents, which can exhibit reduced solubility and slower reaction kinetics in DPPH systems [22]. In addition, background absorbance or matrix-related optical effects in complex extracts may further attenuate apparent radical-scavenging signals. Accordingly, values below LOQ are interpreted as reflecting assay- and matrix-dependent limitations under the applied analytical conditions rather than compositional absence. This observation is consistent with the limited solubility of certain bark-derived phenolics in methanolic systems and the lower sensitivity of the DPPH assay toward high-molecular-weight polyphenols [22].

Recent solvent-based studies further illustrate the variability of DPPH results in *B. vulgaris*. For example, Eroğlu et al. [13] reported DPPH radical inhibition values ranging approximately from 25% to 40% in fruit extracts, whereas ABTS inhibition ranged from 34% to 86%, demonstrating assay-dependent divergence even within the same samples. Vega et al. [19] expressed DPPH activity in mg TE/g DW (76.30 ± 4.00 to 111.37 ± 9.65 mg TE/g DW), corresponding to approximately 305–445 µmol TE/g after conversion. In contrast, Gidic et al. [15] reported DPPH activity as SC_50_ values (0.10–0.14 mg/mL), further emphasizing the diversity of reporting conventions across recent studies.

Because these investigations relied exclusively on solvent extraction without hydrolytic release of bound phenolics, the contribution of non-extractable fractions to radical scavenging capacity may not be fully represented. The present hydrolysis-based approach, targeting total releasable phenolics, therefore provides a complementary perspective for interpreting DPPH activity within a structurally broader phenolic framework.

### 4.6. Phenolic Profile and Its Relation to Antioxidant Assay Responses

Qualitative chromatographic profiling of Berberis vulgaris fruit extracts revealed a structurally diverse phenolic composition, including hydroxybenzoic acids (gallic, syringic), hydroxycinnamic acids (caffeic, chlorogenic), flavan-3-ols (catechin, proanthocyanidin B2), flavonol glycosides (rutin, quercitrin), and the flavonol aglycone quercetin. This profile is consistent with previous HPLC- and LC–MS-based studies on *B. vulgaris* fruits [16]. Several compounds (e.g., chlorogenic acid, caffeic acid, catechin, quercitrin, quercetin) were confirmed by LC–MS, whereas others were tentatively assigned based on HPLC–DAD retention behavior and UV–Vis spectral characteristics, ensuring analytical transparency regarding identification confidence.

In parallel, statistically significant positive correlations were observed between total releasable phenolic content determined after hydrolysis and antioxidant activity measured by FRAP, CUPRAC, and ABTS assays across plant parts. Spearman coefficients ranged from 0.51 to 0.82 in bark and fruits and reached particularly high values in leaves (rs = 0.90–0.95), whereas correlations with DPPH were consistently weaker (rs = 0.33–0.35).

In barberry genotypes analyzed without hydrolysis, Hassanpour and Alizadeh [44] reported statistically significant Pearson correlations between total phenolics and antioxidant assays, with r = 0.471 for DPPH, r = 0.381 for FRAP, and r = 0.526 for Fe^2+^ chelating activity. These coefficients indicate moderate positive associations within solvent-extractable fractions. Likewise, Gidic et al. [15] identified a significant TPC–FRAP relationship (*p* = 0.02) in ultrasonic ethanolic extracts, although the correlation coefficient was not reported and the analysis was limited to a single antioxidant assay. In the present study, TPC–FRAP, TPC–CUPRAC, and TPC–ABTS associations extended into a higher correlation range (rs up to 0.95 in leaves) and were consistently observed across multiple electron-transfer assays. While methodological differences preclude direct numerical comparison, within the analytical framework applied here, TPC demonstrated consistently positive rank-based associations with electron-transfer capacity. Importantly, these correlations indicate an association between bulk phenolic levels and electron-transfer capacity, but they do not imply that total phenolics alone account for the measured antioxidant responses.

The structural classes identified by LC–MS provide mechanistic plausibility for these assay-dependent relationships. Electrochemical investigations have demonstrated that the redox behavior of phenolic acids is strongly influenced by hydroxyl substitution pattern and conjugation [45]. Introduction of an additional hydroxyl group in the aromatic ring lowers oxidation potential and enhances electron-donating capacity. In particular, hydroxycinnamic acids possessing catechol moieties (e.g., caffeic and chlorogenic acids) exhibit improved redox performance due to π-electron delocalization across the aromatic ring and the conjugated side chain [46].

Flavonoid structure–activity relationships further support this interpretation. The presence of an ortho-dihydroxyl (catechol) structure in the B ring, combined with C2=C3 conjugation and a 4-oxo group in the C ring, enhances radical stabilization and electron delocalization [47,48]. Quercetin fulfills these structural criteria (Bors’ criteria) and generally exhibits higher antioxidant activity than its glycosylated derivatives [48]. Glycosylation at the 3-position may reduce planarity and electron delocalization, thereby modulating assay responsiveness [49]. The co-occurrence of quercetin (aglycone), rutin, and quercitrin in the present extracts therefore reflects a structurally heterogeneous flavonoid fraction with potentially differential contributions across assays.

The divergence among antioxidant assays can be rationalized in light of their distinct reaction mechanisms. Electron-transfer-based assays (FRAP, CUPRAC, ABTS) differ in pH and oxidant redox potential [2,48]. FRAP operates under acidic conditions and preferentially detects compounds with sufficiently low oxidation potentials, whereas CUPRAC and ABTS, conducted at near-neutral pH, respond to a broader spectrum of phenolics. In contrast, DPPH reactivity may be more influenced by steric accessibility, solubility, and reaction kinetics, leading to structure-dependent variability among phenolic acids and flavonoids [48]. This mechanistic distinction is consistent with the comparatively weaker TRPC–DPPH correlations observed in the present study.

Taken together, the identified phenolic acids (gallic, chlorogenic, caffeic, syringic), flavan-3-ols (catechin, proanthocyanidin B2), and flavonol derivatives (quercetin, rutin, quercitrin) encompass structural features—multiple hydroxyl groups, catechol moieties, and extended conjugation—recognized as key determinants of redox activity in electrochemical and spectrophotometric systems [45,46,47]. However, given the qualitative scope of chromatographic identification and the absence of quantitative profiling of individual compounds, compound-specific contributions or synergistic interactions cannot be directly inferred. The findings are therefore interpreted within the predefined univariate analytical framework.

### 4.7. Implications for Use of Berberis vulgaris as a Natural Antioxidant Source

Taken together, the results indicate that *B. vulgaris* fruits and leaves represent promising sources of natural antioxidants, with their phenolic content and antioxidant activity influenced by geographical origin. The combined use of complementary antioxidant assays and chromatographic profiling provides a comprehensive evaluation of antioxidant potential, which is essential for the rational selection of plant material for food, nutraceutical, or pharmaceutical applications [1,24].

Several limitations should be acknowledged. First, the Folin–Ciocalteu assay provides an estimate of overall reducing capacity and is not fully specific to phenolic compounds; therefore, non-phenolic reducers (e.g., ascorbic acid and other matrix constituents) may contribute to the reported “total phenolic content” values, and interpretation should be made in conjunction with complementary antioxidant assays and chromatographic evidence [1,6,22]. Second, sampling was conducted within a single growing season and did not explicitly disentangle environmental from genetic effects within sites. Finally, because Lithuania represents a northern European region that is comparatively less studied for *Berberis vulgaris* phytochemistry, these data help fill a geographic gap, but broader multi-year sampling would strengthen generalization and facilitate raw-material selection for practical applications. Within the limitations of the applied assays and sampling design, leaves and fruits emerged as particularly relevant matrices for antioxidant evaluation.

Comparable method- and matrix-dependent divergences between bulk phenolic estimates and radical-scavenging readouts have been reported for phenolic-rich plant extracts, emphasizing that single-assay reporting can misrepresent practical antioxidant performance [22,50]. In addition, multivariate analyses of barberry genotypes based on biochemical constituents and HPLC profiles have highlighted pronounced variability attributable to plant origin, supporting the need to consider geographic and genetic factors when interpreting antioxidant potential [51].

## 5. Conclusions

Although total phenolic content (TPC) did not differ significantly among plant parts when averaged across sites, pronounced site-dependent variability was observed, particularly in fruits, where TPC spanned an approximately three-fold range. Importantly, the use of hydrolysis to assess total releasable phenolics indicates that previously reported solvent-extractable values may underestimate the phenolic pool contributing to antioxidant capacity in this species. While the present study did not separately quantify extractable and bound phenolic fractions, the applied hydrolysis-based protocol enables a more comprehensive estimation of the total phenolic pool potentially contributing to antioxidant behavior. Hydrolysis broadens the phenolic pool but does not mechanically inflate antioxidant outcomes beyond ranges reported in solvent-based studies. Moreover, statistically significant rank-based correlations between total releasable phenolics and electron-transfer assays (FRAP, CUPRAC, ABTS) support the relevance of this broader phenolic pool to measured antioxidant responses within the applied analytical framework.

Antioxidant responses were strongly assay-dependent. Bark exhibited the highest ferric reducing power (FRAP), whereas leaves showed the highest CUPRAC and ABTS activities, and DPPH responses were measurable only in leaf and fruit extracts. These findings confirm that antioxidant capacity in *B. vulgaris* cannot be interpreted as a single intrinsic property but rather reflects the interaction between phenolic structure, reaction mechanism, and analytical conditions. Integration of complementary spectrophotometric assays with chromatographic profiling (HPLC–DAD and LC–MS) enabled interpretation of assay divergence in relation to identified phenolic classes, moving beyond bulk TPC reporting toward structurally informed antioxidant assessment. Given the qualitative scope of chromatographic profiling, structural–assay relationships are interpreted at the level of phenolic classes rather than individual compound quantification and should therefore be regarded as mechanistically plausible rather than quantitatively resolved.

From a broader perspective, the results highlight two important considerations for future research and application: (i) the need to distinguish between solvent-extractable and total releasable phenolic fractions when comparing antioxidant data across studies, and (ii) the importance of geographic context in raw material selection and standardization. In addition, the observed site-dependent variability underscores the potential influence of environmental factors on phenolic abundance and assay responsiveness. By extending phenolic characterization of *B. vulgaris* to northern European populations and incorporating non-extractable fractions within a multi-assay framework, the present study contributes to a more comprehensive understanding of antioxidant behavior in this widely studied yet methodologically heterogeneous species.

## Figures and Tables

**Table 1:** Moisture (loss on drying: LOD) of *Berberis vulgaris* raw materials.

Plant Part	Loss on Drying (%)
Bark	5.5 ± 0.9
Leaves	7.6 ± 0.8
Fruits	9.8 ± 0.2

**Table 2:** Total phenolic content (TPC) in *Berberis vulgaris* extracts by plant part.

Plant Part	TPC (mg GAE/g DW)
Bark	108.7 ± 14.1
Leaves	111.9 ± 8.4
Fruits	121.9 ± 14.7

No statistically significant differences were observed among plant parts (*p* > 0.05).

**Table 3:** Total phenolic content (TPC) by sampling site and plant part.

Site	Bark (mg GAE/g DW)	Leaves (mg GAE/g DW)	Fruits (mg GAE/g DW)
Vilnius (VU Botanical Garden)	109.9 ± 7.0 ^c,d^	111.9 ± 5.0 ^a^	109.2 ± 4.2 ^d,e^
Trakai (Totoriškės)	102.5 ± 9.2 ^b,c^	104.2 ± 16.2 ^b,c^	98.7 ± 3.1 ^c,b^
Kaunas (Girionys)	123.9 ± 9.6 ^e^	166.9 ± 7.8 ^c^	113.4 ± 5.4 ^c,d^
Jonava (Guldynai)	103.5 ± 40.7 ^a,b^	116.8 ± 8.8 ^a^	242.2 ± 61.0 ^e^
Kėdainiai (Paberžė)	123.6 ± 10.2 ^d,e^	81.0 ± 8.1 ^b^	90.3 ± 4.1 ^a,b^
Anykščiai (Pienionys)	88.3 ± 7.8 ^a^	90.5 ± 4.2 ^b^	79.9 ± 5.0 ^a^

Values are presented as mean ± SD for normally distributed variables and as median (IQR) for non-normally distributed variables. Different letters within each column indicate statistically significant differences among sampling sites (*p* < 0.05).

**Table 4:** Antioxidant activity of *Berberis vulgaris* extracts by plant part. Results are expressed as µmol Trolox equivalents per g dry weight (µmol TE/g DW).

Plant Part	FRAP (µmol TE/g DW)	CUPRAC (µmol TE/g DW)	ABTS (µmol TE/g DW)	DPPH (µmol TE/g DW)
Bark	373.2 ± 15.9	248.9 ± 24.5	244.6 ± 27.9	Below LOQ ^1^
Leaves	305.7 ± 32.3	395.7 ± 46.7	346.6 ± 48.5	205.9 ± 84.9
Fruits	275.9 ± 26.7	316.2 ± 32.5	292.4 ± 28.0	243.4 ± 56.6

^1^ LOQ—limit of quantification; “Below LOQ” indicates values below the limit of quantification under the applied analytical conditions and does not imply absence of phenolic or radical-scavenging compounds.

**Table 5:** Phenolic compounds detected in *Berberis vulgaris* fruit extracts by HPLC–DAD and LC–MS.

Compound Class	Phenolic Compound	Detection/Identification Approach	tR (HPLC–DAD, min)	λmax (HPLC–DAD, nm)	tR (LC–MS, min)	Precursor ion [M–H]^−^ (*m*/*z*)
Phenolic acids (hydroxybenzoic and hydroxycinnamic acids)	Gallic acid	Tentatively identified by HPLC–DAD (RT, UV spectrum)	12.64	272	—	—
	Chlorogenic acid	Confirmed by LC–MS (RT, *m*/*z*)	31.12	326	5.35	353
	Caffeic acid	Confirmed by LC–MS (RT, *m*/*z*)	36.05	324	6.03	179
	Syringic acid	Tentatively identified by HPLC–DAD (RT, UV spectrum)	41.71	276–280	—	—
Flavan-3-ols	Catechin	Confirmed by LC–MS (RT, *m*/*z*)	25.71	281	6.75	289
	Proanthocyanidin B2	Tentatively identified by HPLC–DAD (RT, UV spectrum)	29.04	281	—	—
Flavonoid glycoside	Quercitrin (quercetin-3-rhamnoside)	Confirmed by LC–MS (RT, *m*/*z*)	116.37	256, 347	13.47	447
Flavonol glycoside	Rutin (quercetin-3-O-rutinoside)	Tentatively identified by HPLC–DAD (RT, UV spectrum)	97.68	259, 348	—	—
Flavonoid aglycone	Quercetin	Confirmed by LC–MS (RT, *m*/*z*)	145.33	256, 372	18.22	301

Note: *tR*—retention time; λmax—maximum UV absorption wavelength; *m*/*z*—mass-to-charge ratio; [M–H]^−^—deprotonated molecular ion detected in negative ion mode. “–” indicates that the compound was not analyzed by LC–MS and was tentatively identified based on HPLC–DAD retention time and UV spectral data.

**Table 6:** Spearman’s correlation coefficients (rs) between total phenolic content (TPC) (mg GAE/g DW) and antioxidant activity assays in *Berberis vulgaris* extracts.

TPC (mg GAE/g DW) in Plant Parts	FRAP (µmol TE/g DW)	CUPRAC (µmol TE/g DW)	ABTS (µmol TE/g DW)	DPPH (µmol TE/g DW)
	Correlation Coefficient (rs)			
Bark	0.72 *	0.76 *	0.82 *	—
Leaves	0.90 *	0.93 *	0.95 *	0.33 *
Fruits	0.51 *	0.63 *	0.54 *	0.35 *

* Statistically significant correlation at *p* < 0.05.

## Data Availability

The data presented in this study are available on request from the corresponding author.

## Supplementary Materials

The following supporting information can be downloaded at: https://www.mdpi.com/article/10.3390/antiox15030345/s1, Table S1. Ferric reducing antioxidant power (FRAP) by sampling site and plant part. Results are expressed as µmol TE/g DW; Table S2. Cupric reducing antioxidant capacity (CUPRAC) by sampling site and plant part (mean ± SD). Results are expressed as µmol TE/g DW; Table S3. ABTS radical cation scavenging activity by sampling site and plant part. Results are expressed as µmol TE/g DW; Table S4. DPPH radical scavenging activity in leaf and fruit extracts by sampling site. Results are expressed as µmol TE/g DW; Figure S1. Total phenolic content (TPC) by plant part. Boxplots show the distribution of TPC (mg GAE/g DW) in bark, leaves, and fruits. Values represent mean measurements derived from samples collected at six different growing sites; Figure S2. Ferric reducing antioxidant power (FRAP) by plant part. Boxplots illustrate the distribution of FRAP values (µmol TE/g DW) in bark, leaves, and fruits. Data represent measurements from samples collected at six different growing sites; Figure S3. Cupric reducing antioxidant capacity (CUPRAC) by plant part. Boxplots present the distribution of CUPRAC values (µmol TE/g DW) in bark, leaves, and fruits. Data are based on samples collected from six different growing sites; Figure S4. ABTS radical scavenging activity by plant part. Boxplots show the distribution of ABTS values (µmol TE/g DW) in bark, leaves, and fruits. Results are based on samples collected from six different growing sites; Figure S5. Representative LC–MS extracted-ion chromatograms (XICs) and corresponding HPLC–DAD chromatogram of Berberis vulgaris fruit extract illustrating the separation and identification of major phenolic acids (chlorogenic and caffeic acids) and flavonoids (quercetin). Peak assignment was based on retention time and UV–Vis spectra comparison with reference standards; Figure S6. Representative LC–MS extracted-ion chromatograms (XICs) and corresponding HPLC–DAD chromatogram of Berberis vulgaris fruit extract illustrating the identification of flavonoid glycosides and flavan-3-ols. Quercitrin and catechin were identified based on retention time matching and concordant UV–Vis and mass spectral signals obtained using reference standards.
