# Supplementary material for: Site-Dependent Variation in Phenolics and Antioxidant Capacity of Berberis vulgaris Raw Materials: A Multi-Assay Study
Source: Antioxidants (Basel). 2026 Mar 9;15(3):345. doi: 10.3390/antiox15030345 (PMC13023913; doi:10.3390/antiox15030345)
Supplement: Supplementary file 1 [file antioxidants-15-00345-s001.zip › antioxidants-4152560-supplementary.pdf]

Supplementary Materials

Table S1. Ferric reducing antioxidant power (FRAP) by sampling site and plant part. Results are expressed as µmol TE/g DW.

Site	Bark (µmol TE/g DW)	Leaves (µmol TE/g DW)	Fruits (µmol TE/g DW)
Vilnius	399.06 ± 8.4^{B}	298.58 ± 30.8^{c}	313.91 ± 46.1^{c}
Trakai	356.0 ± 11.9^{A}	245.9 ± 82.1^{a,b}	294.4 ± 17.4^{a}
Kaunas	453.5 ± 24.7^{E}	487.7 ± 27.7^{d}	340.8 ± 34.0^{d}
Jonava	276.7 ± 18.2^{A}	343.8 ± 23.9^{c}	256.8 ± 24.4^{b}
Kėdainiai	463.08 ± 20.6^{B}	206.94 ± 15.7^{d}	241.46 ± 19.1^{c}
Anykščiai	291.1 ± 11.5^{E}	251.5 ± 13.7^{a,b}	208.4 ± 19.3^{a}

Data are presented as mean ± SD or median (IQR), as appropriate. Different letters within each column indicate statistically significant differences among sampling sites ($p < 0.05$).

Table S2. Cupric reducing antioxidant capacity (CUPRAC) by sampling site and plant part (mean ± SD). Results are expressed as µmol TE/g DW.

Site	Bark (µmol TE/g DW)	Leaves (µmol TE/g DW)	Fruits (µmol TE/g DW)
Vilnius	273.3 ± 22.4^{B}	382.5 ± 43.4^{b,d}	354.8 ± 25.4^{b}
Trakai	215.1 ± 20.7^{A}	360.4 ± 68.5^{a,b}	333.8 ± 52.8^{a}
Kaunas	307.3 ± 51.2^{A}	586.2 ± 58.6^{d}	403.5 ± 27.4^{b}
Jonava	203.3 ± 14.8^{B}	444.2 ± 45.6^{c}	301.5 ± 28.1^{c}
Kėdainiai	295.2 ± 15.8^{A}	268.5 ± 32.8^{b,c}	271.8 ± 36.1^{b,c}
Anykščiai	199.3 ± 22.1^{B}	332.3 ± 31.4^{a}	232.0 ± 25.0^{a}

Data are presented as mean ± SD or median (IQR), as appropriate. Different letters within each column indicate statistically significant differences among sampling sites ($p < 0.05$).

Table S3. ABTS radical cation scavenging activity by sampling site and plant part. Results are expressed as µmol TE/g DW.

Site	Bark (µmol TE/g DW)	Leaves (µmol TE/g DW)	Fruits (µmol TE/g DW)
Vilnius	282.1 ± 29.5^{B}	356.9 ± 38.4^{b,d}	342.8 ± 20.3^{b}
Trakai	214.3 ± 20.2^{A}	249.6 ± 29.9^{a,b}	331.6 ± 18.2^{a}
Kaunas	295.0 ± 48.7^{A}	564.0 ± 53.9^{d}	374.9 ± 4.3^{b}
Jonava	186.6 ± 21.2^{B}	392.0 ± 49.7^{c}	268.7 ± 2.5^{c}
Kėdainiai	299.2 ± 33.8^{A}	247.6 ± 27.1^{b,c}	237.2 ± 14.5^{b,c}
Anykščiai	191.2 ± 13.7^{B}	266.5 ± 28.6^{a}	196.8 ± 15.4^{a}

Data are presented as mean ± SD or median (IQR), as appropriate. Different letters within each column indicate statistically significant differences among sampling sites ($p < 0.05$).

Table S4. DPPH radical scavenging activity in leaf and fruit extracts by sampling site. Results are expressed as µmol TE/g DW.

Site	Leaves (µmol TE/g DW)	Fruits (µmol TE/g DW)
Vilnius	259.4 ± 118.8^{A}	$251.8 \pm 72.2^{a,b}$
Trakai	204.5 ± 96.4^{A}	$268.7 \pm 66.8^{a,b}$
Kaunas	269.5 ± 153.4^{A}	308.7 ± 55.2^{b}
Jonava	193.8 ± 71.5^{A}	231.9 ± 58.6^{a}
Kėdainiai	170.9 ± 41.3^{A}	242.7 ± 53.1^{a}
Anykščiai	137.5 ± 27.9^{A}	156.8 ± 33.6^{c}

Data are presented as mean ± SD or median (IQR), as appropriate. Different letters within each column indicate statistically significant differences among sampling sites ($p < 0.05$). **Note:** DPPH activity in bark extracts was below the limit of quantification (LOQ) and is therefore not reported.

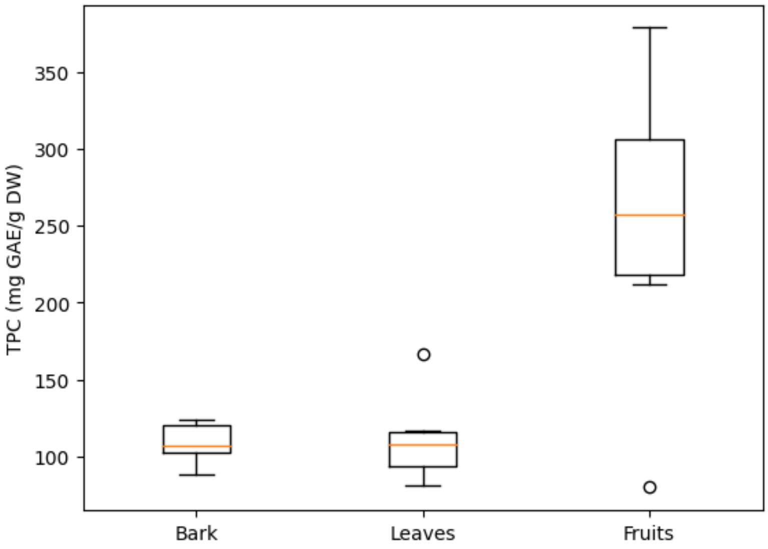

Figure S1. Total phenolic content (TPC) by plant part. Boxplots show the distribution of TPC (mg GAE/g DW) in bark, leaves, and fruits. Values represent mean measurements derived from samples collected at six different growing sites.

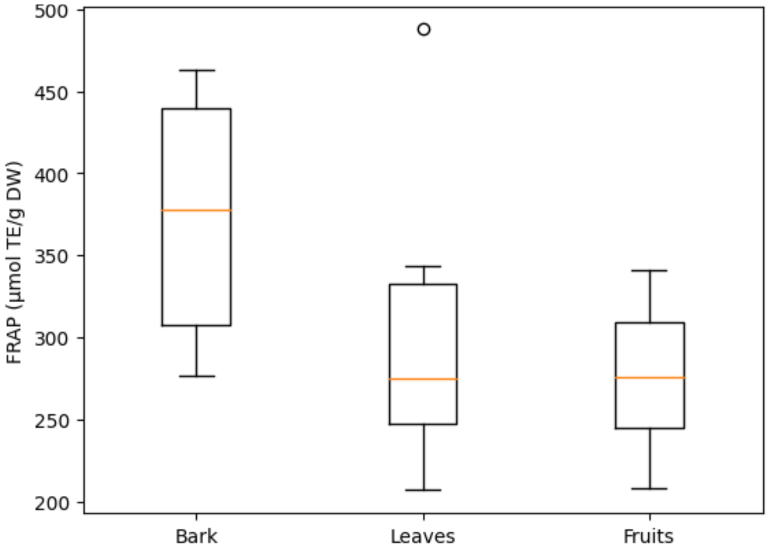

Figure S2. Ferric reducing antioxidant power (FRAP) by plant part. Boxplots illustrate the distribution of FRAP values (µmol TE/g DW) in bark, leaves, and fruits. Data represent measurements from samples collected at six different growing sites.

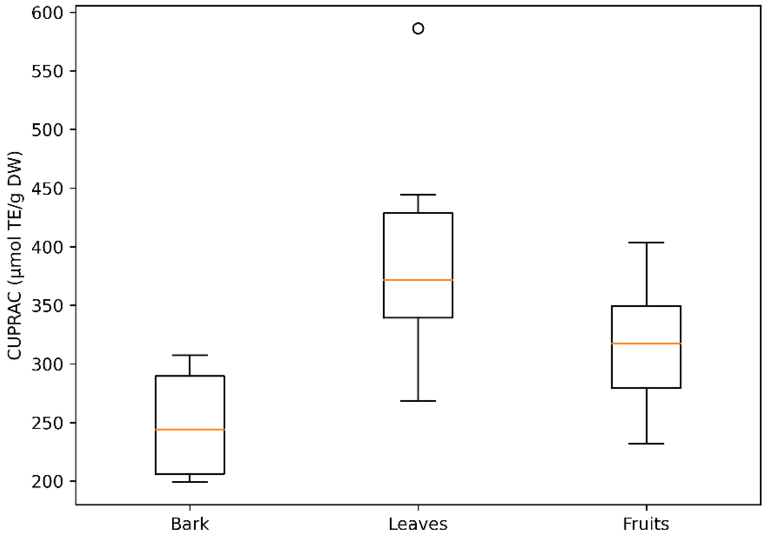

Figure S3. Cupric reducing antioxidant capacity (CUPRAC) by plant part. Boxplots present the distribution of CUPRAC values (µmol TE/g DW) in bark, leaves, and fruits. Data are based on samples collected from six different growing sites.

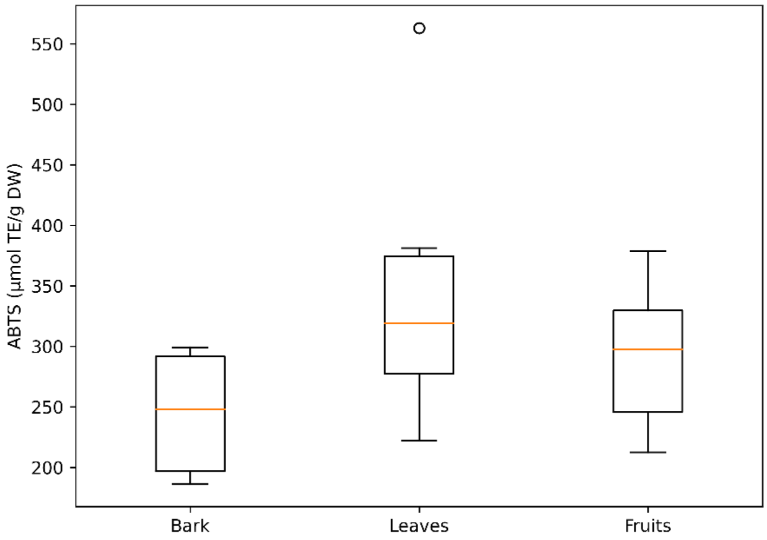

Figure S4. ABTS radical scavenging activity by plant part. Boxplots show the distribution of ABTS values (µmol TE/g DW) in bark, leaves, and fruits. Results are based on samples collected from six different growing sites.

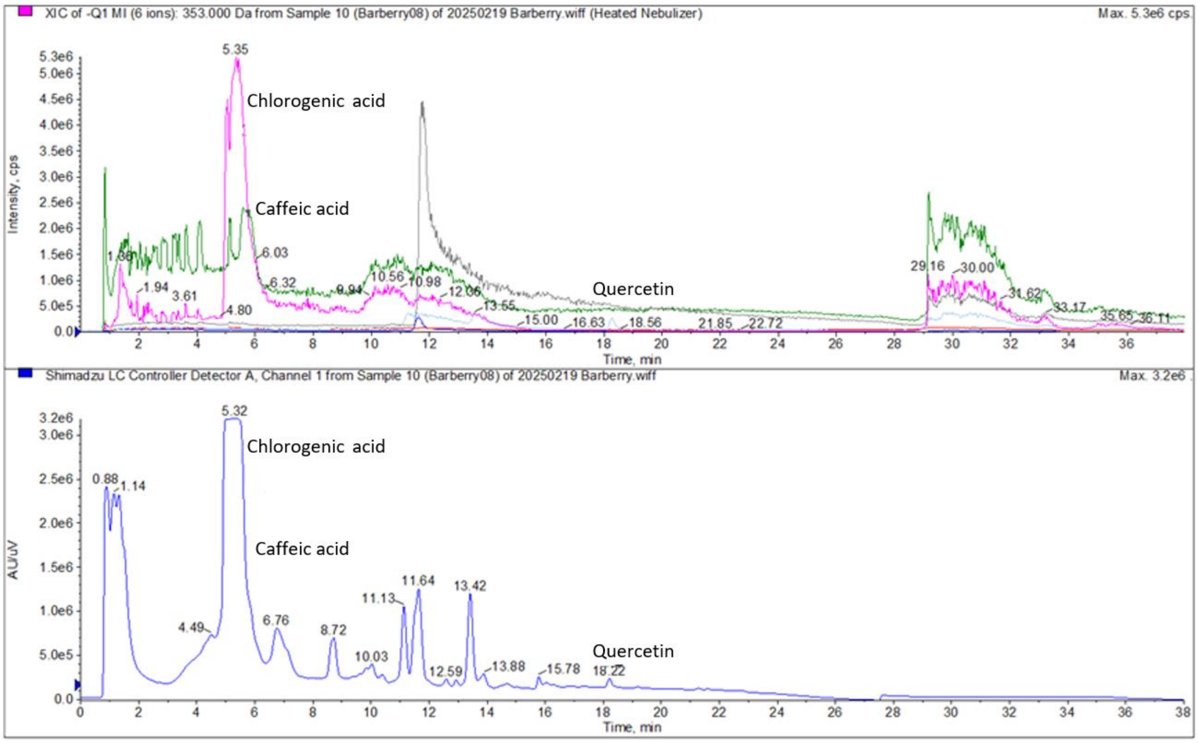

Figure S5. Representative LC–MS extracted-ion chromatograms (XICs) and corresponding HPLC–DAD chromatogram of *Berberis vulgaris* fruit extract illustrating the separation and identification of major phenolic acids (chlorogenic and caffeic acids) and flavonoids (quercetin). Peak assignment was based on retention time and UV–Vis spectra comparison with reference standards.

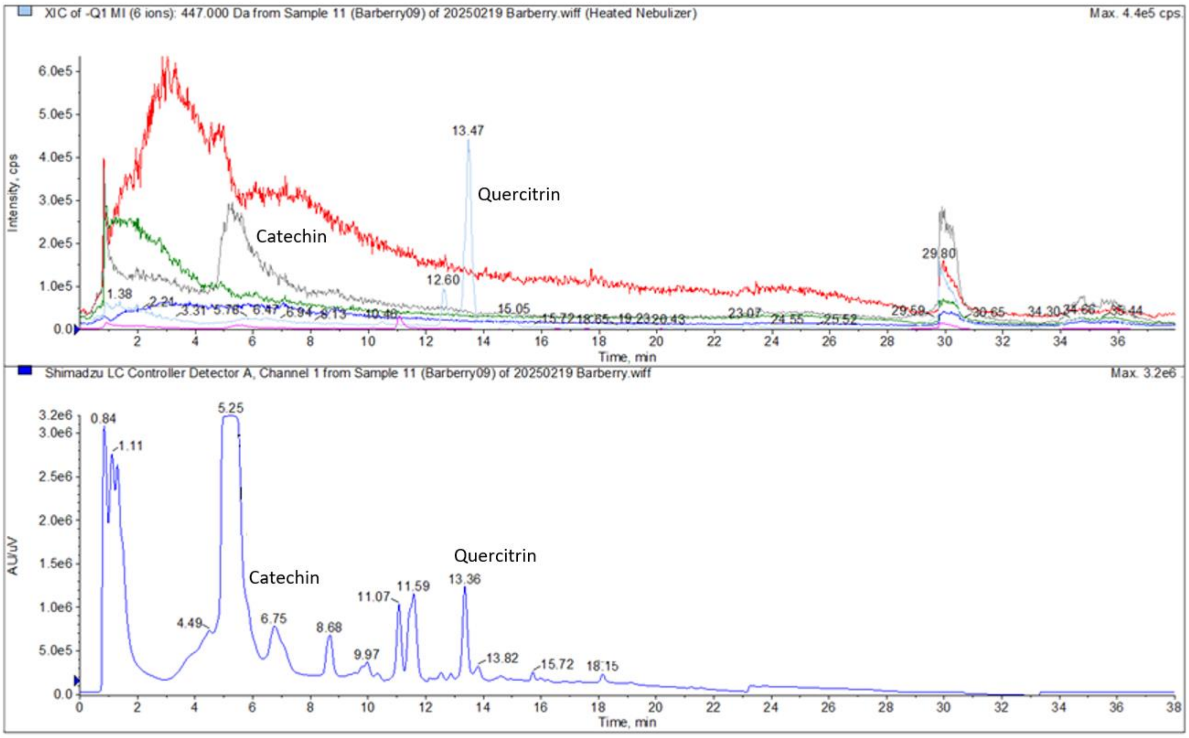

Figure S6. Representative LC–MS extracted-ion chromatograms (XICs) and corresponding HPLC–DAD chromatogram of *Berberis vulgaris* fruit extract illustrating the identification of flavonoid glycosides and flavan-3-ols. Quercitrin and catechin were identified based on retention time matching and concordant UV–Vis and mass spectral signals obtained using reference standards.
